# Differential Interaction of Platelet-Derived Extracellular Vesicles With Circulating Immune Cells: Roles of TAM Receptors, CD11b, and Phosphatidylserine

**DOI:** 10.3389/fimmu.2018.02797

**Published:** 2018-12-11

**Authors:** Birgit Fendl, Tanja Eichhorn, René Weiss, Carla Tripisciano, Andreas Spittler, Michael B. Fischer, Viktoria Weber

**Affiliations:** ^1^Christian Doppler Laboratory for Innovative Therapy Approaches in Sepsis, Department for Biomedical Research, Danube University Krems, Krems, Austria; ^2^Core Facility Flow Cytometry & Surgical Research Laboratories, Medical University of Vienna, Vienna, Austria

**Keywords:** extracellular vesicles, immune cells, TAM receptors, CD11b, flow cytometry, phosphatidylserine

## Abstract

Secretion and exchange of biomolecules by extracellular vesicles (EVs) are crucial in intercellular communication and enable cells to adapt to alterations in their microenvironment. EVs are involved in a variety of cellular processes under physiological conditions as well as in pathological settings. In particular, they exert profound effects on the innate immune system, and thereby are also capable of modulating adaptive immunity. The mechanisms underlying their interaction with their recipient cells, particularly their preferential association with monocytes and granulocytes in the circulation, however, remain to be further clarified. Surface molecules exposed on EVs are likely to mediate immune recognition and EV uptake by their recipient cells. Here, we investigated the involvement of Tyro3, Axl, and Mer (TAM) tyrosine kinase receptors and of integrin CD11b in the binding of platelet-derived EVs, constituting the large majority of circulating EVs, to immune cells in the circulation. Flow cytometry and Western Blotting demonstrated a differential expression of TAM receptors and CD11b on monocytes, granulocytes, and lymphocytes, as well as on monocyte subsets. Of the TAM receptors, only Axl and Mer were detected at low levels on monocytes and granulocytes, but not on lymphocytes. Likewise, CD11b was present on circulating monocytes and granulocytes, but remained undetectable on lymphocytes. Differentiation of monocytes into classical, intermediate, and non-classical monocyte subsets revealed distinct expression patterns of Mer and activated CD11b. Co-incubation of isolated monocytes and granulocytes with platelet-derived EVs showed that the binding of EVs to immune cells was dependent on Ca^++^. Our data do not support a particular role for TAM receptors or for activated CD11b in the association of platelet-derived EVs with monocytes and granulocytes in the circulation, as anti-TAM antibodies did not interfere with EV binding to isolated immune cells, as binding was not dependent on the presence of TIM4 acting synergistically with TAM receptors, and as neither low levels of Gas6, required as a linker between phosphatidylserine (PS) on the EV surface and TAM receptors on immune cells, nor masking of PS on the EV surface did interfere with EV binding.

## Introduction

Extracellular vesicles (EVs) are important intercellular transmitters of biological information due to their ability to shuttle biologically active molecules, such as lipids, peptides, ribonucleic acids, or sugars between cells. EVs can influence a variety of cellular functions and contribute to intercellular communication in a multitude of physiological as well as pathological conditions, including inflammation, autoimmune disorders, and immunosuppression following trauma or progressive sepsis (1–3).

EVs released by both, immune cells (monocytes, granulocytes, and lymphocytes) and non-immune cells (endothelial cells, platelets, red blood cells) can exert critical effects on the innate immune response and are also capable of modulating adaptive immunity by mediating the cross-talk between cells of the innate and adaptive immune systems. Depending on their cells of origin, their membrane properties, and their cargo (4–6), EVs can either enhance or suppress the immune response by delivering pathogen- or tumor-derived antigens to antigen-presenting cells (7, 8), by exposing pro-apoptotic molecules (9, 10), or via the transfer of bioactive factors including microRNAs (11, 12) and eicosanoids (13) to their target cells. A number of studies have provided evidence that EVs interact with immune cells in the circulation, where they are primarily associated with monocytes and granulocytes (4, 6, 14, 15). EVs can induce the release of neutrophil extracellular traps (16), amplifying autoimmune reactions, or aggravating inflammation and thrombosis. EV-induced immune regulation has been proposed to depend on cell surface signaling (17) or on the incorporation of EVs by immune cells (4), but the mechanisms underlying EV-immune cell interaction as well as the functional consequences of EV binding or uptake by immune cells remain to be further elucidated (18, 19).

Cellular uptake of EVs is strongly inhibited at 4°C, indicating an energy-dependent process, and treatment of either EVs or recipient cells with proteinase K reduces EV uptake, compatible with receptor-mediated mechanisms (20, 21). Only few specific cellular receptors for EVs have, however, been proposed to date, including integrins (22, 23) as well as T cell immunoglobulin mucin (TIM) receptors, which have been implicated in the binding to phosphatidylserine (PS) exposed on the EV surface (24). EV uptake may not only depend on specific receptors, but might require adaptor or bridging molecules secreted by recipient or donor cells, or by other cells in the microenvironment. Heparan sulfate exposed on the plasma membrane has been reported to function as EV receptor in a mechanism in which fibronectin acts as a bridging molecule (25, 26), and the chemokine CCL18 can act as a linker between glycans exposed on the EV surface and cancer cells (27). Likewise, members of the Tyro3, Axl, and Mer (TAM) receptor family can mediate endothelial uptake of platelet-derived EVs in the presence of growth arrest-specific molecule 6 (Gas6) or protein S as bridging molecules (28).

The TAM receptors, homologous type I receptor tyrosine kinases, are activated by their vitamin K-dependent ligands Gas6 and protein S (29, 30), which bind to PS exposing moieties via their N-terminal γ-carboxyglutamic acid domains (GLA domains). Several signaling functions of TAM receptors in homeostasis and inflammation have been described, including a critical role in the homeostatic clearance of apoptotic cells (31–33). TAM receptor expression has been reported in many cell types (31), including antigen-presenting cells, natural killer cells, and monocytes (34, 35). Mer, named for its expression on monocytes, endothelial cells, and the reproductive system (36), has been described as the most prominent TAM receptor in the immune system, but little information exists on the constitutive TAM receptor expression in human peripheral blood. Here, we examined the expression of TAM receptors and of integrin CD11b on circulating lymphocytes, granulocytes, monocytes, and monocyte subsets and provide evidence that none of these receptors is solely capable of mediating the binding of EVs to circulating monocytes and granulocytes. We further show that the binding of EVs to immune cells is strictly dependent on Ca^++^, while masking of PS does not interfere with EV binding.

## Materials and Methods

### Human Blood and Platelet Concentrates

Whole blood was collected by aseptic venipuncture from healthy volunteer donors into vacuum tubes (Vacuette, Greiner Bio-One, Kremsmuenster, Austria) containing ethylene diamine tetraacetic acid (EDTA) using a 21 gauge needle. Blood collection was approved by the Review Board of Danube University Krems, and written informed consent was obtained from all donors. Platelet concentrates for EV isolation were obtained from the Clinic for Blood Group Serology and Transfusion Medicine, Medical University Vienna, Austria, after approval by the Ethics Committee (ECS2177/2013). They were produced in a blood bank setting from healthy individuals eligible for single donor platelet apheresis using a Trima Accel^®;^ automated blood collection system (Version 5.0, Terumo BCT, Lakewood, CO), stored in polyolefin bags in SSP^+^ solution (Macopharma, Tourcoing, France) at a ratio of 80% SSP^+^ and 20% plasma, and were used for EV isolation within 1–3 days.

### Cell Culture Media and Reagents

Phosphate buffered saline (PBS) and PBS^−/−^ without calcium and magnesium (Life Technologies, Paisley, UK) were filtered through 0.1 μm filters (Merck Millipore, Billerica, MA) before use. RPMI-1640 (RPMI), Medium 199 (M199), 4-(2-hydroxyethyl)-1-piperazineethanesulfonic acid (HEPES), penicillin-streptomycin, and ethylenediaminetetraacetic acid disodium salt (EDTA) were purchased from Sigma Aldrich (St. Louis, MO). RPMI was supplemented with 10% vesicle-depleted AB serum (centrifuged at 20,000 g for 30 min at 4°C and sterile filtered) where indicated. M199 was supplemented with 20 mM HEPES, 100 IU/ml penicillin, 100 μg/ml streptomycin, 15 IU/ml unfractionated heparin (Gilvasan Pharma, Vienna, Austria), 10 μg/ml endothelial cell growth supplement (BD Biosciences, Bedford, MA), and 20% fetal bovine serum (FBS, heat inactivated at 56°C for 30 min, sterile filtered, Sigma Aldrich). Reagents for sodium dodecyl sulfate polyacrylamide gel electrophoresis (SDS-PAGE) and Western Blotting, including gels (4–20%, Mini-PROTEAN), molecular weight markers (Precision plus Western C protein standard), and membranes (Trans-Blot transfer pack, nitrocellulose), were obtained from Bio-Rad (Hercules, CA). Gas6 was quantified in AB serum and in isolated EV fractions using a human Gas6 enzyme linked immunosorbent assay (R&D Systems, Minneapolis, MN). All antibodies used for flow cytometry, blocking assays, confocal microscopy, and Western Blotting are specified in Table S1.

### Monocyte and Granulocyte Isolation

Freshly drawn whole blood (20 ml) was layered onto Polymorphprep gradient centrifugation medium (20 ml, Alere Technologies AS, Oslo, Norway) to separate granulocytes and peripheral blood mononuclear cells (PBMCs) from red blood cells by centrifugation at 500 g for 30 min at room temperature (RT). The cell suspension containing granulocytes and PBMCs (15 ml) was layered on Ficoll-Paque PLUS (30 ml, GE Healthcare, Uppsala, Sweden) and centrifuged at 400 g for 20 min at RT to separate PBMCs and granulocytes. Monocytes were isolated from PBMCs by negative depletion of non-monocytes via indirect magnetic labeling (Pan Monocyte Isolation Kit, Miltenyi Biotec, Bergisch Gladbach, Germany). The amount of residual red blood cells and platelets in monocyte and granulocyte preparations was determined by blood cell counting (Sysmex KX-21 N, Sysmex, Neumuenster, Germany), and monocyte and granulocyte purity was additionally characterized by flow cytometry after staining with CD14-PE and CD45-PB for monocytes and with CD66b-APC for granulocytes.

### Isolation and Characterization of Extracellular Vesicles

EVs were isolated from platelet concentrates generated by single donor platelet apheresis, using differential centrifugation as previously described (37). EV fractions were characterized using flow cytometry, imaging flow cytometry, cryo-electron microscopy, and nanoparticle tracking analysis (37). Briefly, after removal of platelets by centrifugation (2,500 g, 15 min) at RT, EVs were pelleted at 20,000 g (30 min, 4°C) using a Sorvall Evolution RC ultracentrifuge, Rotor SS-34 (Thermo Fisher Scientific, Waltham, MA). The pellet was washed with sterile PBS^−/−^, re-centrifuged at 20,000 g (30 min, 4°C), re-suspended in PBS^−/−^ to a final protein concentration of 4 mg/ml (DC Protein Assay, Bio-Rad), aliquoted, and stored at −80°C until further use. Centrifugation of the 20,000 g supernatant at 100,000 g (Optima MAX ultracentrifuge, MLA-80 rotor, Beckman Coulter) yielded EV fraction II (37). All experiments on the interaction of EVs with immune cells were carried out using EV fraction I, while EV fraction II was only used for comparison of phenotypic marker expression by Western Blotting as described below.

### Flow Cytometric Characterization of Extracellular Vesicles

EVs (fraction I) were diluted 400-fold in PBS^−/−^ and stained with FITC-conjugated lactadherin as marker of PS as well as with CD41-PC7 as marker of platelet origin. Staining was performed for 15 min in the dark, and all antibodies were centrifuged at 17,000 g for 10 min before use. Stained samples were diluted 5-fold in PBS^−/−^, and analyzed on a Gallios flow cytometer (Beckman Coulter, Brea, CA) equipped with 405 nm, 488 nm, and 638 nm lasers. Fluorescent-green silica particles (1, 0.5, 0.3 μm; excitation/emission 485/510 nm; Kisker Biotech, Steinfurt, Germany) were used for calibration, the triggering signal was set to forward scatter/size, and the EV gate was set below the 1 μm bead cloud as previously described (37) and as shown in Figure S1. Data were acquired for 3 min at a flow rate of 30 μl/min and analyzed using the Kaluza Software (Beckman Coulter). Buffer controls, isotype controls, and single stainings of specific monoclonal antibodies are shown in Figure S1.

### Characterization of Extracellular Vesicle Fractions by Western Blotting

EV fractions I and II were characterized for the expression of CD63 and CD81 (tetraspanins), Alix (apoptotic linked-gene-product 2 interacting protein X; involved in endosomal sorting), and αActinin-1 (cytoskeletal protein) by Western Blotting. Aliquots of EV fractions I and II (20 μg protein per lane) were loaded onto polyacrylamide gels, separated by SDS-PAGE under reducing or non-reducing conditions (CD63), respectively, blotted onto nitrocellulose, and incubated with monoclonal antibodies against CD63 (final concentration 1 μg/ml), CD81 (2 μg/ml), Alix (0.5 μg/ml), and αActinin-1 (0.5 μg/ml). Membranes were developed using the Western Breeze chemiluminescent kit (Invitrogen, Carlsbad, CA).

### CD11b, TIM4, and TAM Receptor Expression on Immune Cells in Whole Blood

Aliquots of 200 μl of freshly drawn whole blood were stained with CD14-PE, CD16-PC5, CD66b-APC, and CD45-PB for 15 min in the dark to identify immune cell populations including monocyte subsets. To assess the surface expression of activated CD11b, Axl, and Mer on immune cells, CD11b-FITC, Axl-AF488, or MERTK-PC7 were included in the staining protocol. To characterize Tyro3 or TIM4 surface expression, Tyro3/Dtk-PE or TIM4-PE were added, and CD14-PE was replaced by CD14-APC AF750. After staining, blood samples were treated with 2 ml FACS Lysing Solution (BD Biosciences, Franklin Lakes, NJ) for 10 min to lyse red blood cells, remaining cells were pelleted for 5 min at 500 g, washed and re-suspended in 500 μl PBS^−/−^, and analyzed on a Gallios flow cytometer (Beckman Coulter). Gating was performed by exclusion of platelets and remaining red blood cells based on their lack of CD45 expression. Granulocytes were excluded based on their CD66b expression, lymphocytes were identified as CD45^+^CD14^−^ cells, and their exclusion allowed for the identification of monocytes as previously described (15). Monocyte subsets were further discriminated based on their CD14 and CD16 expression into classical monocytes (CM; CD14^++^CD16^−^), intermediate monocytes (IM; CD14^++^CD16^+^), and non-classical monocytes (NCM; CD14^+^CD16^++^) as previously described (15, 38) and shown in Figure S2.

### CD11b, TIM4, and TAM Receptor Expression on Isolated Monocytes and Granulocytes

Isolated monocytes and granulocytes were resuspended in PBS^−/−^ at a concentration of 5 x 10^5^ cells in 100 μl. Staining and flow cytometric analysis was performed as described in section CD11b, TIM4, and TAM Receptor Expression on Immune Cells in Whole Blood above.

### TAM Receptor Expression Analysis on Isolated Granulocytes and Monocytes by Western Blotting

Freshly isolated granulocytes or monocytes were washed in ice-cold PBS^−/−^ and lysed by incubation (30 min, 4°C) with RIPA buffer (25 mM Tris pH 7.6, 150 mM NaCl, 1% NP-40, 1% sodium deoxycholate, 0.1% SDS; Cell Biolabs, San Diego, CA) supplemented with a protease inhibitor cocktail (complete, Roche, Basel, Switzerland) according to the instructions of the supplier. The lysate was centrifuged for 10 min at 14,000 g, the supernatant was collected, and protein was quantified prior to aliquoting and storage at −80°C until further use. For SDS-PAGE, aliquots of the cell lysates corresponding to 10 μg protein were loaded per lane. Gels were run under reducing conditions, blotted onto nitrocellulose, incubated with anti-Tyro3, anti-Axl, and anti-Mer antibodies at a final concentration of 1 μg/ml, and developed using the Western Breeze chemiluminescent kit (Invitrogen). Human umbilical vein endothelial cells (HUVECs), which have previously been shown to express Axl and Mer (28, 36, 39), were used as positive control.

### Effect of Ca^++^, Phosphatidylserine, CD11b, and TAM Receptors on the Binding of Extracellular Vesicles to Granulocytes and Monocytes

**Figure 5:**
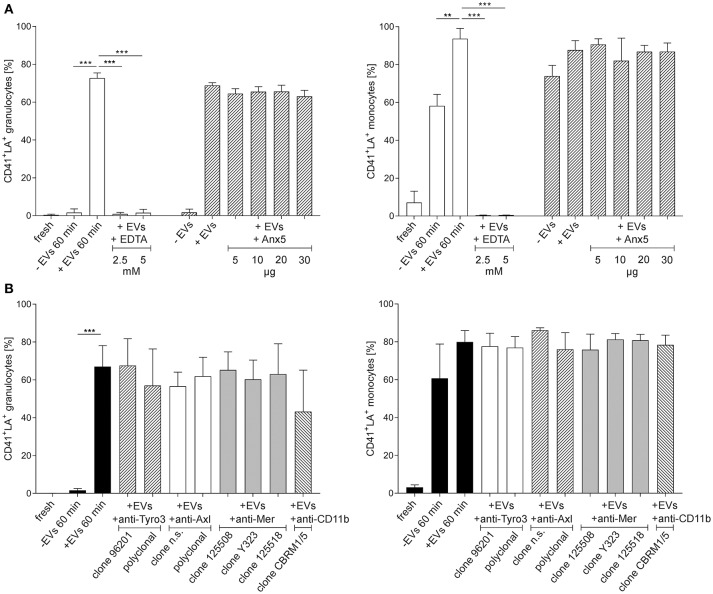
Effect of Ca^++^, phosphatidylserine, TAM receptors, and CD11b on the binding of platelet-derived extracellular vesicles to granulocytes and monocytes. Isolated granulocytes and monocytes were incubated with platelet-derived EVs as described in the Methods section, or left untreated. Granulocytes or monocytes associated with platelet-derived EVs were identified as CD41^+^lactadherin^+^ events in flow cytometry. Characterization was performed in freshly isolated immune cells (fresh) and after co-incubation of immune cells with platelet-derived EVs (+EVs 60 min). Immune cells incubated without EVs served as control (–EVs 60 min). The different levels of granulocytes or monocytes associated with EVs in the control at 60 min are due to endogenous EV release from residual platelets. As specified in the Results section, platelets were hardly detectable in isolated granulocytes, while isolated monocytes contained significant amounts of residual platelets, resulting in EV release during incubation. **(A)** To assess the dependence of the interaction on Ca^++^, co-incubation of EVs and immune cells was performed both in the presence and absence of EDTA (2.5 and 5 mM final concentration). To mask PS, platelet-derived EVs were pre-incubated with Anx5, and the efficiency of PS blocking was confirmed by the abrogation of thrombin generation in the presence of Anx5 as shown in Figure S5. **(B)** The influence of TAM receptor or CD11b blocking was assessed by pre-treatment of granulocytes and monocytes with anti-Tyro3, anti-Axl, anti-Mer, or anti-CD11b antibodies as specified in Table S1, followed by co-incubation of the pre-treated immune cells with platelet-derived EVs, revealing that neither TAM receptor blockade nor masking of activated CD11b had an impact on the binding of platelet-derived EVs to granulocytes and monocytes (*n* = 3). Data are given as mean ± standard deviation. **p < 0.01; ^***^p < 0.001. LA, lactadherin; n.s., not specified.

Freshly isolated granulocytes and monocytes were seeded into 24-well suspension culture plates (Greiner Bio-One) at a density of 250,000 cells per well in a total volume of 800 μl RPMI containing 10% vesicle-depleted AB serum, resulting in a final concentration of Gas6 of 2.7 ng/ml. To explore the influence of activated CD11b or TAM receptors on the binding of EVs, cells were incubated with anti-CD11b (25 μg/ml) (40), anti-Tyro3, anti-Axl, or anti-Mer antibodies (clones as specified in Figure 5B and Table S1; 5 μg/ml each) (28) for 10 min at 37°C, or left untreated. Platelet-derived EVs were added at a protein concentration of 25 μg/ml, corresponding to approximately 40 EVs per immune cell, and incubation was continued for 60 min. Cell suspensions were harvested, cells were pelleted by centrifugation (300 g, 10 min), re-suspended in 100 μl PBS^−/−^, and their association with EVs was determined using flow cytometry (see above) and confocal microscopy. To assess the dependence of EV binding to immune cells on Ca^++^, incubation of isolated granulocytes and monocytes with platelet-derived EVs was performed both in the presence and absence of EDTA (2.5 and 5 mM final concentration). To mask PS, platelet-derived EVs (20 μg protein) were pre-incubated with 5, 10, 20, and 30 μg annexin V for 30 min (Anx5; BioLegend, San Diego, CA), and the efficiency of blocking was confirmed by the abrogation of thrombin generation in the presence of Anx5 as described below.

### Thrombin Generation

To confirm the masking of PS on EVs by pre-incubation with Anx5, the ability of Anx5-treated and untreated EVs to support thrombin generation was assessed. Platelet-derived EVs (25 μg protein/ml) were pre-incubated with 0.1, 1, 10, or 25 μg/ml Anx5 for 30 min, or left untreated. The EV suspensions were added to vesicle-free human plasma, and thrombin generation was assessed using a thrombin generation assay (Technoclone, Vienna, Austria) based on the thrombin-dependent cleavage of a fluorogenic substrate over time (37). Measurements were recorded at 37°C for 60 min with 1 min intervals using a Synergy 2 reader (Bio-Tek Instruments Inc., Winooski, VT) at 360/460 nm, and data were analyzed with the Bio-Tek Gen5 software (*n* = 2).

### Confocal Microscopy

Freshly isolated granulocytes and monocytes were stained for 15 min with lactadherin-AF647, CD45-PB, CD41-AF488, and either CD66b-PE for granulocytes or CD14-PE for monocytes. Stained cells were transferred to adhesion slides (Marienfeld, Lauda-Königshofen, Germany) and incubated for 30 min at RT to allow for adherence. Cells were fixed with 4% formaldehyde (Thermo Scientific, Rockford, IL) for 15 min, washed with PBS, and mounted with Fluoromount aqueous mounting medium (Sigma Aldrich). Images were acquired at excitation wavelengths of 405, 488, 561, and 633 nm using a TCS SP8 confocal laser scanning microscope (Leica, Mannheim, Germany) equipped with a 63x objective (numerical aperture 1.3). Image analysis was performed using the LAS X software (Leica).

### Statistical Analysis

Statistical analysis was performed using GraphPad Prism version 7.02 (La Jolla, CA). Data are presented as mean ± standard deviation. For comparison of two groups of normally distributed data, paired *t*-test or unpaired *t*-test with or without Welch's correction was used. For not-normally distributed data, Wilcoxon matched pairs signed rank test (paired) or Mann-Whitney test (unpaired) was used. For multiple comparisons of normally distributed data, repeated measures one-way ANOVA followed by Bonferroni's multiple comparisons test was used while Friedman test followed by Dunn's multiple comparisons test was used for not-normally distributed data. Numbers of independent experiments are indicated in the Figure legends. Significance was accepted at *p* < 0.05.

## Results

### CD11b and TAM Receptors Are Differentially Expressed on Monocytes, Granulocytes, and Lymphocytes as Well as on Monocyte Subsets

**Figure 1:**
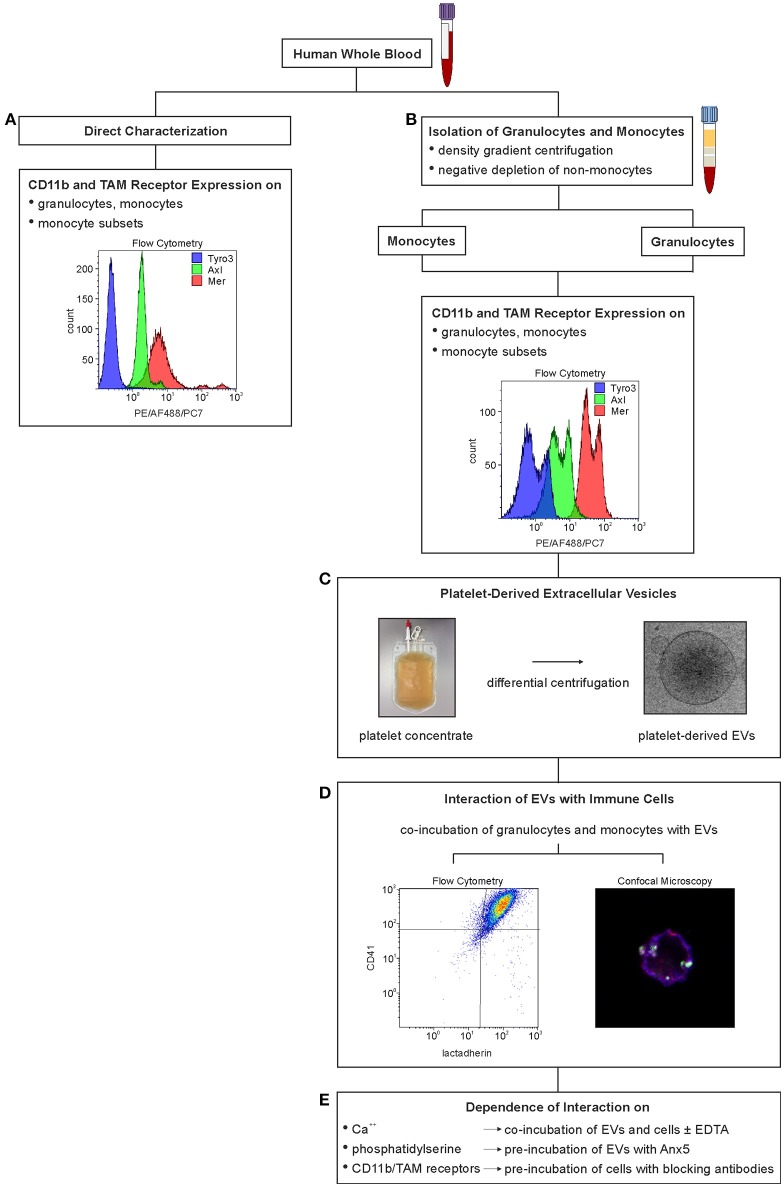
Experimental set-up to characterize the interaction of immune cells with platelet-derived extracellular vesicles. Expression of TAM receptors and of activated CD11b on immune cells was either characterized directly in whole blood using flow cytometry **(A)** or on isolated granulocytes and monocytes **(B)**. Platelet-derived EVs were isolated from medical grade platelet concentrates **(C)**, and their interaction with immune cells was investigated by co-incubation with granulocytes and monocytes isolated from whole blood **(D)**. The effect of Ca^++^, phosphatidylserine, activated CD11b, and TAM receptors on the interaction of EVs and immune cells was assessed by addition of EDTA, by pre-incubation of platelet-derived EVs with Anx5, and by pre-incubation of isolated immune cells with anti-CD11b and anti-TAM antibodies, respectively **(E)**.

**Figure 2:**
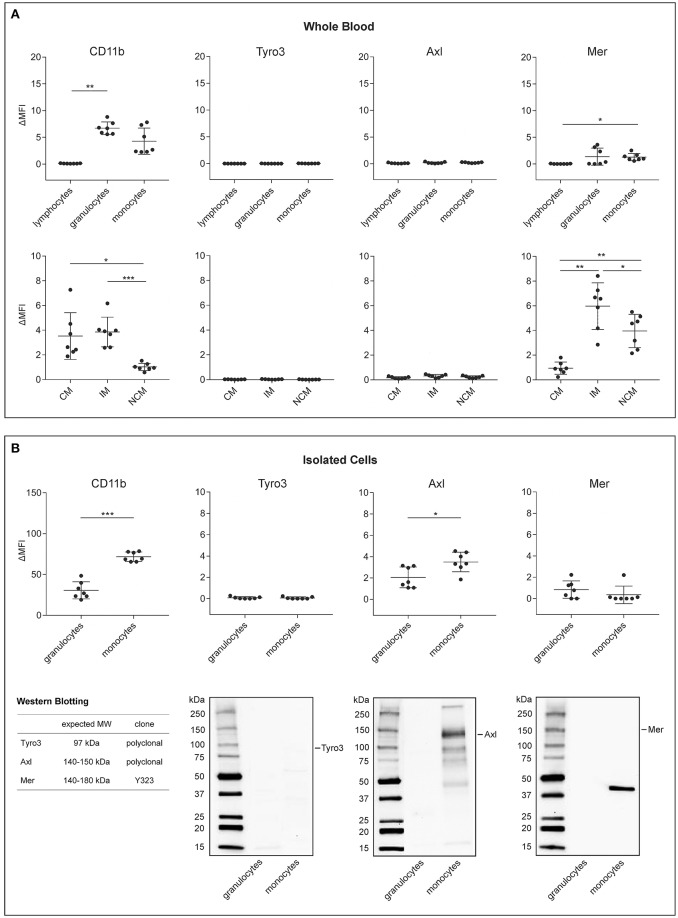
Expression of CD11b and TAM receptors on leukocytes and monocyte subsets in human whole blood and on isolated immune cells. The surface expression of activated CD11b, Tyro3, Axl, and Mer was assessed directly in whole blood on **(A)** lymphocytes, granulocytes, and monocytes (upper panel) as well as on classical, intermediate, and non-classical monocytes (CM, IM, and NCM, lower panel) using flow cytometry (*n* = 7). The flow cytometric characterization of monocyte subsets in whole blood and in isolated monocyte preparations is shown in Figure S2. **(B)** Expression of activated CD11b and TAM receptors on isolated granulocytes and monocytes using flow cytometry (*n* = 7). Data are given as mean ± standard deviation. *p < 0.05; ^**^p < 0.01; ^***^p < 0.001. TAM receptor expression on isolated granulocytes and monocytes using Western Blotting are shown in the lower panel. Ten microgram of protein were loaded per lane. The band detected for Mer does not correspond to full-length Mer, which has an expected molecular mass of 140–180 kDa, as indicated in the Figure. ΔMFI refers to the difference in mean fluorescence intensity between the fluorochrome-labeled specific antibodies and the corresponding isotype controls. MW, molecular mass.

We initially assessed the expression of activated CD11b and of TAM receptors on immune cells using flow cytometry, since members of the TAM receptor family, particularly Axl and its ligand Gas6, have recently been shown to mediate the phagocytosis of platelet-derived EVs by human endothelial cells (28), and since CD11b has been implicated in the uptake of red blood cell-derived EVs by monocytes (23). TAM receptor and CD11b surface expression were characterized on lymphocytes, granulocytes, and monocytes directly in whole blood and on freshly isolated granulocytes and monocytes as summarized in Figure 1 and shown in detail in Figure 2A for whole blood and in Figure 2B for isolated granulocytes and monocytes. Tyro3 remained undetectable on immune cells under both conditions. Axl was not detectable on immune cells in whole blood, but was found on isolated granulocytes and monocytes, albeit at low levels, and Mer was found to be weakly expressed on granulocytes and monocytes both in whole blood and after isolation, while it was not detectable on lymphocytes. Western Blotting of lysates from isolated granulocytes and monocytes confirmed the absence of Tyro3 on granulocytes and monocytes. Low levels of Axl were detected on isolated monocytes, while no signal was obtained for granulocytes, and full-length Mer remained undetectable in Western Blots of both granulocytes and monocytes. TAM receptor expression on HUVEC was analyzed as control and revealed the presence of Axl and of low levels of Mer in consistence with published findings (28), as shown in Figure S3. Activated CD11b was detected by flow cytometry on granulocytes and monocytes in whole blood and after isolation, but was not expressed on lymphocytes. Differentiation of monocytes into classical, intermediate, and non-classical monocytes as shown in Figure S2 revealed a distinct expression of activated CD11b, with comparable levels on classical and intermediate monocytes, but significantly lower expression on non-classical monocytes. Likewise, Mer was differentially expressed on monocyte subsets, with low levels on classical monocytes, but significantly higher expression on intermediate and non-classical monocytes. TIM4, which has been implicated in the tethering of PS-exposing apoptotic cells to macrophages, remained undetectable on circulating immune cells (data not shown).

### Phenotypic Characterization of Extracellular Vesicle Fractions Reveals Distinct Protein Patterns

**Figure 3:**
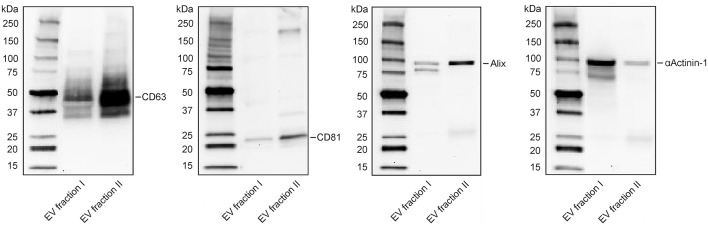
Characterization of extracellular vesicle fractions by Western Blotting. Detection of CD63, CD81, Alix, and αActinin-1 in EV fraction I (enriched in microvesicles) and EV fraction II (enriched in exosomes). Twenty microgram protein were loaded per lane. Exposure time: 5 min for CD63, 11 min for CD81, 10 min for Alix, and 4 min for αActinin-1. Expected bands: 30–60, 22–26, 96, 100 kDa, respectively.

Isolation of platelet-derived EVs from platelet concentrates yielded two distinct vesicle populations, of which EV fraction I (pelleted at 20,000 g) was enriched in microvesicles, and EV fraction II (pelleted by centrifugation of the 20,000 g supernatant at 100,000 g), was enriched in exosomes. EV fraction I, which was used for all further experiments in this study, contained 90% CD41^+^lactadherin^+^ events in the EV gate according to flow cytometry (37). Its size distribution according to nanoparticle tracking analysis (NTA) is shown in Figure S4. Western Blotting revealed distinct patterns for CD63, CD81, Alix, and αActinin-1 for EV fraction I and II. αActinin-1, which has recently been identified as a reliable marker for enrichment of microvesicles (41), was predominantly found in EV fraction I, while the exosome markers CD63, CD81, and Alix were strongly enriched in fraction II (Figure 3).

### Platelet-Derived Extracellular Vesicles Bind to Granulocytes and Monocytes

**Figure 4:**
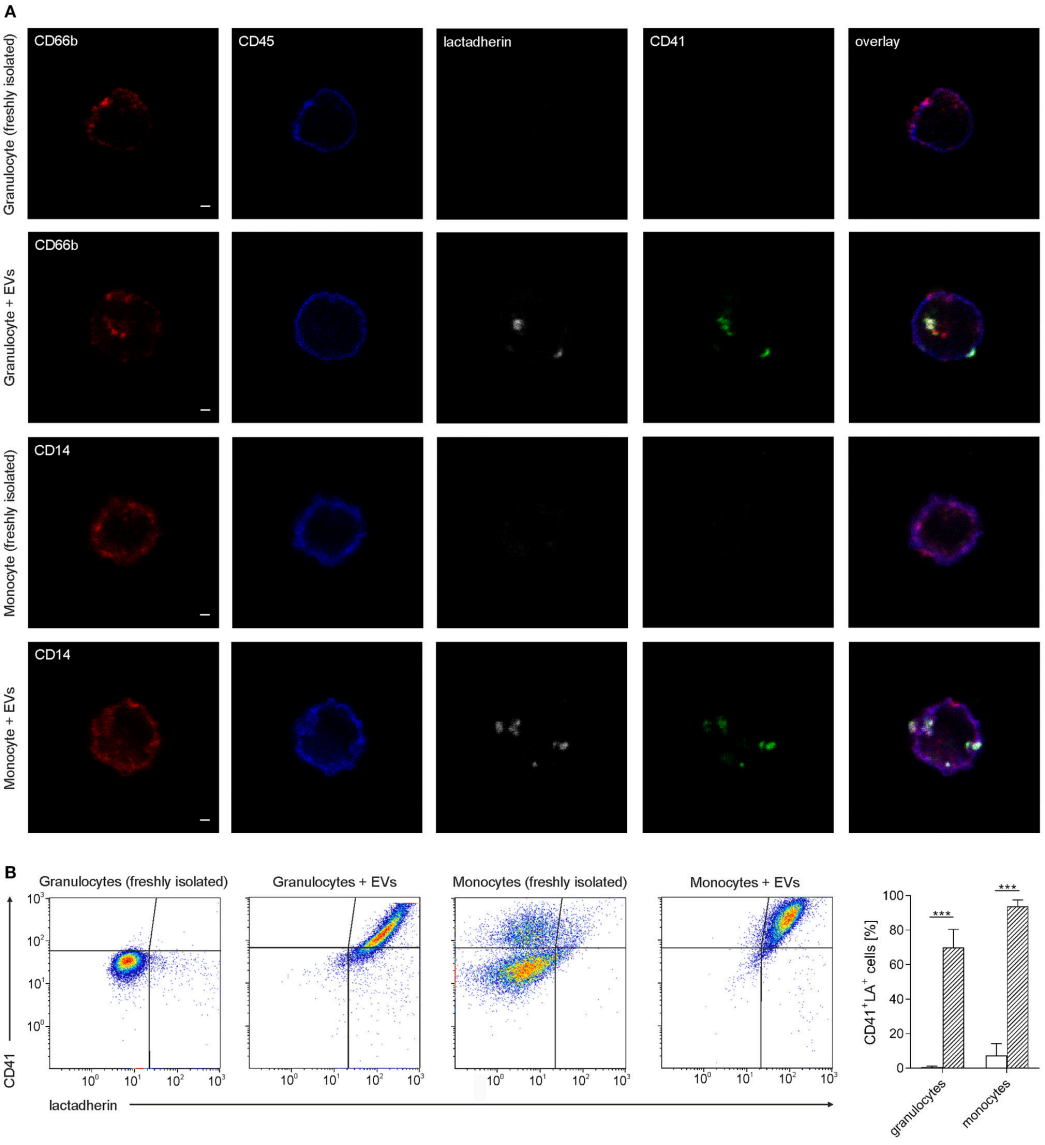
Association of granulocytes and monocytes with platelet-derived extracellular vesicles. Granulocytes and monocytes were incubated with platelet-derived EVs as described in the Methods section, using a ratio of cells to EVs of approximately 1:40. Staining with CD45-PB (leukocyte marker; blue), CD66b-PE (granulocyte marker; red), CD14-PE (monocyte marker; red), CD41-AF488 (platelet marker, green), and lactadherin-AF647 (marker for PS exposing EVs; white) was performed either directly after granulocyte and monocyte isolation or after co-incubation of granulocytes and monocytes with platelet-derived EVs. **(A)** Stained cells were fixed on adhesion slides and analyzed by confocal microscopy. Representative confocal images of granulocytes and monocytes with or without co-incubation with EVs are shown. In the overlay, platelet-derived EVs appear as white/green dots. Scale bars, 1 μm. **(B)** Granulocytes and monocytes associated with platelet-derived EVs were identified as CD41^+^lactadherin^+^ cells using flow cytometry. Representative CD41 vs. lactadherin scatter plots for granulocytes, granulocytes incubated with EVs, monocytes, and monocytes incubated with EVs are shown from left to right. The bar chart summarizes the percentages of CD41^+^lactadherin^+^ granulocytes and monocytes (i.e., granulocytes and monocytes associated with platelet-derived EVs) in freshly isolated immune cells (white bars) and after incubation with platelet-derived EVs (hatched bars, *n* = 7). Data are given as mean ± standard deviation. ^***^p < 0.001.

Granulocyte preparations contained 51.8 ± 4.8% CD66b^+^ cells, and red blood cells constituted the large majority of residual cells, while platelets were hardly detectable (*n* = 7). Isolated monocyte preparations contained 77.2 ± 11.2% CD45^+^CD14^+^ cells, and the residual cells were identified as platelets according to flow cytometry (*n* = 7). Immediately after isolation, 0.6 ± 0.5% of all granulocytes and 7.3 ± 6.9% of all monocytes were associated with platelet-derived EVs (*n* = 7). Co-incubation of isolated granulocytes and monocytes with platelet-derived EVs, using a ratio of approximately 40 EVs per cell, resulted in efficient binding of EVs to both, granulocytes and monocytes, with 70 ± 1.4% EV-granulocyte aggregates (CD41^+^lactadherin^+^ granulocytes) and 93.8 ± 3.7% EV-monocyte aggregates (CD41^+^lactadherin^+^ monocytes) after 60 min, as shown in Figure 4. Incubation of granulocytes and monocytes without addition of EVs (control) did not result in significant changes of EV-granulocyte aggregates (1.4 ± 1.4% after 60 min), while EV-monocyte aggregates increased significantly to 53.2 ± 16.1% after 60 min, indicating the release of EVs from residual platelets and their binding to monocytes.

### Binding of Extracellular Vesicles to Granulocytes and Monocytes is Dependent on Ca^++^, but Does Not Require Phosphatidylserine

Next, we proceeded to investigate whether the binding of platelet-derived EVs to granulocytes and monocytes would require Ca^++^ and PS (Figure 5A). Co-incubation of granulocytes and monocytes with platelet-derived EVs in the presence of EDTA completely abrogated the binding of EVs to both, granulocytes and monocytes. Pre-incubation of EVs with increasing concentrations of Anx5 to mask PS, however, had no influence on the subsequent binding of EVs to granulocytes and monocytes. Thrombin generation assays showed that pre-treatment of EVs with Anx5 reduced thrombin generation in a dose-dependent manner and resulted in complete suppression of thrombin generation at higher levels of Anx5, confirming the efficient masking of PS on the EV surface (Figure S5), and indicating that binding of EVs to immune cells is not dependent on PS.

### Binding of Extracellular Vesicles to Granulocytes and Monocytes is not Inhibited by Blockade of TAM Receptors or CD11b

To explore the potential involvement of TAM receptors or activated CD11b in the binding of platelet-derived EVs to granulocytes and monocytes, we investigated the effect of TAM receptor and CD11b blockade as shown in Figure 5B, using granulocytes and monocytes that were pre-incubated with different anti-TAM receptor antibodies or with anti-CD11b as specified in Table S1. None of the antibodies used in the blocking experiments reduced EV binding to granulocytes and monocytes, indicating that neither TAM receptors nor activated CD11b are primarily involved in the adhesion of platelet-derived EVs to granulocytes or monocytes.

## Discussion

Circulating EVs represent a large interactive surface capable of establishing contacts with cells or with molecules in the cellular microenvironment (42). The functional significance of surface molecules for targeting EVs to their recipient cells is illustrated by the manifold interactions of EVs with immune cells, including EV-mediated antigen presentation (7, 43), EV-mediated induction of apoptosis (44, 45), and the contribution of innate immune cell-derived EVs to the modulation of adaptive immunity (46).

Tetraspanins, integrins, lectins, proteoglycans, as well as components of the extracellular matrix have been implicated in mediating the contact of EVs with their target cells (19, 47), but the mechanisms underlying the differential interaction of EVs with immune cells in the circulation remain to be further elucidated. There is evidence that circulating EVs are preferentially associated with monocytes and granulocytes (4, 14, 15), which can affect EV-induced immune regulation as well as homeostatic clearance processes. The rapid, efficient, and immunologically silent phagocytic removal of excess, damaged, or aged cells and of EVs released from these cells is vital, since uncleared cellular debris has been linked to a variety of pathologies, including autoimmune diseases.

In the current study, we characterized the surface expression of Tyro3, Axl, and Mer tyrosine kinase receptors and of activated CD11b on circulating immune cells and examined their involvement in the binding of platelet-derived EVs to isolated monocytes and granulocytes. The EVs used in this study were derived from non-activated platelets and represented an EV population enriched in PS-exposing microvesicles. We chose to focus on the role of TAM receptors, as their requirement for the constant clearance of PS-exposing apoptotic cells is well established (48–51). This prompted us to assume that similar mechanisms might mediate the interaction of PS-exposing EVs with immune cells and that differences in TAM receptor expression on circulating monocytes, granulocytes, and lymphocytes might result in the preferential binding of PS-exposing EVs to distinct immune cell populations. In support of this hypothesis, Axl has recently been shown to mediate endothelial uptake of platelet-derived EVs (28) in a Gas6 dependent-manner, and although little information exists on the constitutive expression of TAM receptors in human peripheral whole blood, evidence has been provided for a differential expression of TAM receptors on circulating leukocytes (31, 36). Moreover, analysis of TAM receptor expression in patients suffering from systemic lupus erythematosus (SLE) has convincingly indicated increased expression of Mer on CD14^++^CD16^+^ intermediate monocytes (36).

Our findings substantiate that the binding of EVs to immune cells is dependent on Ca^++^, as the addition of EDTA completely blocked the binding of platelet-derived EVs to isolated monocytes and granulocytes. Since this requirement for Ca^++^, a typical feature of GLA domain-dependent interactions with negatively charged phospholipids, was compatible with an involvement of TAM receptors in EV-immune cell interaction, we initially assessed TAM receptor expression on the main leukocyte populations by flow cytometric analysis directly in whole blood to minimize any potential influence of cellular activation during immune cell isolation. Our findings confirmed the expression of Mer on circulating monocytes from healthy donors, while none of the leukocyte populations expressed significant levels of Axl or Tyro3. Differentiation of monocytes into classical, intermediate, and non-classical subsets provided clear evidence for increased expression of Mer in the CD16^+^ monocyte subsets, particularly in CD14^++^CD16^+^ intermediate monocytes. This is in agreement with published data on differential Mer expression on monocyte subsets in SLE patients (36) as well as with previous findings from our own laboratory showing a preferential association of platelet-derived EVs with intermediate monocytes in freshly drawn whole blood (15). While the three subsets represent a continuous line of monocyte development in the circulation, with classical monocytes giving rise to intermediate and non-classical monocytes (52, 53), functional differences exist between these subpopulations. CD16^+^ monocytes have been reported to preferentially phagocytose apoptotic cells (54), and the increased expression of Mer on CD16^+^ monocyte subsets would thus seem consistent with a role for Mer in the uptake of PS-exposing EVs. Contrary to this hypothesis, however, pre-incubation of isolated monocytes or granulocytes with anti-Mer antibodies did not result in reduced binding of platelet-derived EVs to either monocytes or granulocytes. Moreover, Gas6, required as linker between PS exposed on EVs and Mer, was present at about 10-fold lower levels in our samples as compared to previous studies (28), as we used only 10% human serum in the co-incubation experiments. These low Gas6 concentrations did, however, not impede the binding of EVs by isolated immune cells, adding further evidence that the EV-immune cell interaction did not primarily depend on Mer. It could be argued that the interaction of PS-exposing EVs to monocytes and granulocytes might require initial TIM4-mediated tethering of EVs to the cell surface prior to Mer-mediated EV uptake, as established for the engulfment of PS-exposing apoptotic cells by macrophages (50, 55). Still, isolated monocytes readily bound platelet-derived EVs in our study, while they did not express TIM4, contradicting a role for TIM4 in tethering of platelet-derived EVs to immune cells. Finally, our data do not support a crucial role of PS for the binding of platelet-derived EVs to immune cells, since pre-incubation of EVs with Anx5 did not reduce their binding even under conditions where PS was fully masked, as substantiated by the complete abrogation of PS-dependent thrombin generation in control experiments. In line with these findings, previous studies have provided evidence that Anx5, even when present at high molar excess, failed to inhibit the binding of Gas6 to apoptotic cells (33) or to platelet-derived EVs (28), suggesting that membrane microdomains, rather than general exposure of PS, might be relevant for the binding of Gas6 and other PS ligands to the EV surface.

In addition to investigating the role of TAM receptors, we assessed the involvement of the integrin CD11b on EV binding to immune cells, as several studies have provided indications for a role of integrins in the binding and uptake of EVs. CD11b, in particular, has been implicated in the uptake of red blood cell-derived EVs by monocytes (23), and platelet factor 4 (PF4) has recently been identified as a ligand for the integrin Mac-1 (CD11b/CD18, αMβ2, complement receptor 3), suggesting a role of PF4 as platelet-derived alarmin (56). While this might imply a function for CD11b/CD18 in the binding of platelet-derived EVs expressing PF4, CD11b blocking experiments did not confirm a significant involvement of CD11b in EV binding.

To conclude, binding and uptake of EVs by immune cells can either serve apoptotic EV clearance, which is crucial in maintaining homeostasis in all multicellular organisms, or can mediate specific functions of immune cells. Our current data, on the whole, do not support a particular role for TAM receptors or for activated CD11b in the association of platelet-derived EVs with monocytes and granulocytes in the circulation, since (i) anti-TAM or anti-CD11b antibodies did not interfere with binding, (ii) binding was not dependent on the presence of TIM4, which has been shown to act synergistically with TAM receptors, and (iii) neither low levels of Gas6, nor (iv) masking of PS on the EV surface did impede EV binding.

Clearly, the functional implications of EV association with immune cells are of particular interest. The EV populations used in this study were found to trigger the dose-dependent release of IL-8 from monocytic THP-1 cells as well as from primary human monocytes (Figure S6). Starting from these findings, one important aspect in our further investigations will be whether EVs from healthy donors and from inflammatory settings exhibit comparable effects and to elucidate whether the immunologically silent clearance of EVs and specific EV-mediated immunomodulatory processes are based on the same mechanisms.

## Author Contributions

BF characterized the interaction of EVs with isolated immune cells, analyzed the data, and wrote the paper together with VW. RW performed flow cytometric characterization of immune cells and EVs. TE performed confocal microscopic analysis of immune cell-EV interaction. CT performed TAM receptor and EV marker expression experiments using Western Blotting and thrombin generation assays. MF contributed to experimental design and data interpretation. AS supported the characterization of EVs by flow cytometry and contributed to data analysis. VW conceived and coordinated the study, contributed to data interpretation, and wrote the manuscript together with BF and TE. All authors analyzed the results and approved the final version of the manuscript.

### Conflict of Interest Statement

The authors declare that the research was conducted in the absence of any commercial or financial relationships that could be construed as a potential conflict of interest.

## References

[1] Yuana Y Sturk A Nieuwland R. Extracellular vesicles in physiological and pathological conditions. Blood Rev. (2013) 27:31–9. 10.1016/j.blre.2012.12.002 23261067

[2] Turpin D Truchetet ME Faustin B Augusto JF Contin-Bordes C Brisson A. Role of extracellular vesicles in autoimmune diseases. Autoimmun Rev. (2016) 15:174–83. 10.1016/j.autrev.2015.11.004 26554931

[3] Karasu E Eisenhardt SU Harant J Huber-Lang M. Extracellular Vesicles: Packages Sent With Complement. Front Immunol. (2018) 9:721. 10.3389/fimmu.2018.00721 29696020 PMC5904200

[4] Di Trapani M Bassi G Midolo M Gatti A Kamga PT Cassaro A. Differential and transferable modulatory effects of mesenchymal stromal cell-derived extracellular vesicles on T, B and NK cell functions. Sci Rep. (2016) 6:24120. 10.1038/srep24120 27071676 PMC4829861

[5] Borger V Bremer M Ferrer-Tur R Gockeln L Stambouli O Becic A. Mesenchymal stem/stromal cell-derived extracellular vesicles and their potential as novel immunomodulatory therapeutic agents. Int J Mol Sci. (2017) 18:E1450. 10.3390/ijms18071450 28684664 PMC5535941

[6] Robbins PD Morelli AE. Regulation of immune responses by extracellular vesicles. Nat Rev Immunol. (2014) 14:195–208. 10.1038/nri3622 24566916 PMC4350779

[7] Segura E Guerin C Hogg N Amigorena S Thery C. CD8+ dendritic cells use LFA-1 to capture MHC-peptide complexes from exosomes *in vivo*. J Immunol. (2007) 179:1489–96. 10.4049/jimmunol.179.3.1489 17641014

[8] Tran TH Mattheolabakis G Aldawsari H Amiji M. Exosomes as nanocarriers for immunotherapy of cancer and inflammatory diseases. Clin Immunol. (2015) 160:46–58. 10.1016/j.clim.2015.03.021 25842185

[9] Gregory CD Dransfield I. Apoptotic tumor cell-derived extracellular vesicles as important regulators of the onco-regenerative niche. Front Immunol. (2018) 9:1111. 10.3389/fimmu.2018.01111 29875772 PMC5974173

[10] Koncz G Hancz A Chakrabandhu K Gogolak P Kerekes K Rajnavolgyi E. Vesicles released by activated T cells induce both Fas-mediated RIP-dependent apoptotic and Fas-independent nonapoptotic cell deaths. J Immunol. (2012) 189:2815–23. 10.4049/jimmunol.1102827 22891283

[11] Alexander M Hu R Runtsch MC Kagele DA Mosbruger TL Tolmachova T. Exosome-delivered microRNAs modulate the inflammatory response to endotoxin. Nat Commun. (2015) 6:7321. 10.1038/ncomms8321 26084661 PMC4557301

[12] Wang X Gu H Qin D Yang L Huang W Essandoh K. Exosomal miR-223 contributes to mesenchymal stem cell-elicited cardioprotection in polymicrobial sepsis. Sci Rep. (2015) 5:13721. 10.1038/srep13721 26348153 PMC4562230

[13] Boilard E. Extracellular vesicles and their content in bioactive lipid mediators: more than a sack of microRNA. J Lipid Res. (2018) 59:2037–46. 10.1194/jlr.R084640 29678959 PMC6210911

[14] Fendl B Weiss R Fischer MB Spittler A Weber V. Characterization of extracellular vesicles in whole blood: influence of pre-analytical parameters and visualization of vesicle-cell interactions using imaging flow cytometry. Biochem Biophys Res Commun. (2016) 478:168–73. 10.1016/j.bbrc.2016.07.073 27444383

[15] Weiss R Groger M Rauscher S Fendl B Eichhorn T Fischer MB. Differential interaction of platelet-derived extracellular vesicles with leukocyte subsets in human whole blood. Sci Rep. (2018) 8:6598. 10.1038/s41598-018-25047-x 29700367 PMC5920058

[16] Dieker J Tel J Pieterse E Thielen A Rother N Bakker M. Circulating apoptotic microparticles in systemic lupus erythematosus patients drive the activation of dendritic cell subsets and prime neutrophils for NETosis. Arthritis Rheumatol. (2016) 68:462–72. 10.1002/art.39417 26360137

[17] Muller L Simms P Hong CS Nishimura MI Jackson EK Watkins SC. Human tumor-derived exosomes (TEX) regulate Treg functions via cell surface signaling rather than uptake mechanisms. Oncoimmunology (2017) 6:e1261243. 10.1080/2162402X.2016.1261243 28919985 PMC5593709

[18] Prada I Meldolesi J. Binding and Fusion of extracellular vesicles to the plasma membrane of their cell targets. Int J Mol Sci. (2016) 17:E1296. 10.3390/ijms17081296 27517914 PMC5000693

[19] Mulcahy LA Pink RC Carter DR. Routes and mechanisms of extracellular vesicle uptake. J Extracell Vesicles (2014) 3:24641. 10.3402/jev.v3.24641 25143819 PMC4122821

[20] Escrevente C Keller S Altevogt P Costa J. Interaction and uptake of exosomes by ovarian cancer cells. BMC Cancer (2011) 11:108. 10.1186/1471-2407-11-108 21439085 PMC3072949

[21] Paggetti J Haderk F Seiffert M Janji B Distler U Ammerlaan W. Exosomes released by chronic lymphocytic leukemia cells induce the transition of stromal cells into cancer-associated fibroblasts. Blood (2015) 126:1106–17. 10.1182/blood-2014-12-618025 26100252 PMC4560344

[22] Jethwa SA Leah EJ Zhang Q Bright NA Oxley D Bootman MD. Exosomes bind to autotaxin and act as a physiological delivery mechanism to stimulate LPA receptor signalling in cells. J Cell Sci. (2016) 129:3948–57. 10.1242/jcs.184424 27557622 PMC5087657

[23] Straat M van Hezel ME Boing A Tuip-De Boer A Weber N Nieuwland R. Monocyte-mediated activation of endothelial cells occurs only after binding to extracellular vesicles from red blood cell products, a process mediated by beta-integrin. Transfusion (2016) 56:3012–20. 10.1111/trf.13851 27933619

[24] Miyanishi M Tada K Koike M Uchiyama Y Kitamura T Nagata S. Identification of Tim4 as a phosphatidylserine receptor. Nature (2007) 450:435–9. 10.1038/nature06307 17960135

[25] Christianson HC Svensson KJ van Kuppevelt TH Li JP Belting M. Cancer cell exosomes depend on cell-surface heparan sulfate proteoglycans for their internalization and functional activity. Proc Natl Acad Sci USA. (2013) 110:17380–5. 10.1073/pnas.1304266110 24101524 PMC3808637

[26] Purushothaman A Bandari SK Liu J Mobley JA Brown EE Sanderson RD. Fibronectin on the surface of myeloma cell-derived exosomes mediates exosome-cell interactions. J Biol Chem. (2016) 291:1652–63. 10.1074/jbc.M115.686295 26601950 PMC4722448

[27] Berenguer J Lagerweij T Zhao XW Dusoswa S van der Stoop P Westerman B. Glycosylated extracellular vesicles released by glioblastoma cells are decorated by CCL18 allowing for cellular uptake via chemokine receptor CCR8. J Extracell Vesicles (2018) 7:1446660. 10.1080/20013078.2018.1446660 29696074 PMC5912193

[28] Happonen KE Tran S Morgelin M Prince R Calzavarini S Angelillo-Scherrer A. The Gas6-Axl protein interaction mediates endothelial uptake of platelet microparticles. J Biol Chem. (2016) 291:10586–601. 10.1074/jbc.M115.699058 27006397 PMC4865908

[29] Stitt TN Conn G Gore M Lai C Bruno J Radziejewski C. The anticoagulation factor protein S and its relative, Gas6, are ligands for the Tyro 3/Axl family of receptor tyrosine kinases. Cell (1995) 80:661–70. 10.1016/0092-8674(95)90520-0 7867073

[30] Varnum BC Young C Elliott G Garcia A Bartley TD Fridell YW. Axl receptor tyrosine kinase stimulated by the vitamin K-dependent protein encoded by growth-arrest-specific gene 6. Nature (1995) 373:623–6. 10.1038/373623a0 7854420

[31] van der Meer JH van der Poll T van 't Veer C. TAM receptors, Gas6, and protein S: roles in inflammation and hemostasis. Blood (2014) 123:2460–9. 10.1182/blood-2013-09-528752 24596417

[32] Geng K Kumar S Kimani SG Kholodovych V Kasikara C Mizuno K. Requirement of gamma-carboxyglutamic acid modification and phosphatidylserine binding for the activation of Tyro3, Axl, and mertk receptors by growth arrest-specific 6. Front Immunol (2017) 8:1521. 10.3389/fimmu.2017.01521 29176978 PMC5686386

[33] Dransfield I Zagorska A Lew ED Michail K Lemke G. Mer receptor tyrosine kinase mediates both tethering and phagocytosis of apoptotic cells. Cell Death Dis. (2015) 6:e1646. 10.1038/cddis.2015.18 25695599 PMC4669813

[34] Seitz HM Camenisch TD Lemke G Earp HS Matsushima GK. Macrophages and dendritic cells use different Axl/Mertk/Tyro3 receptors in clearance of apoptotic cells. J Immunol. (2007) 178:5635–42. 10.4049/jimmunol.178.9.5635 17442946

[35] Graham DK Dawson TL Mullaney DL Snodgrass HR Earp HS. Cloning and mRNA expression analysis of a novel human protooncogene, c-mer. Cell Growth Differ. (1994) 5:647–57. 8086340

[36] Hilliard BA Zizzo G Ulas M Linan MK Schreiter J Cohen PL. Increased expression of Mer tyrosine kinase in circulating dendritic cells and monocytes of lupus patients: correlations with plasma interferon activity and steroid therapy. Arthritis Res Ther. (2014) 16:R76. 10.1186/ar4517 24650765 PMC4060208

[37] Tripisciano C Weiss R Eichhorn T Spittler A Heuser T Fischer MB. Different potential of extracellular vesicles to support thrombin generation: contributions of phosphatidylserine, tissue factor, and cellular origin. Sci Rep. (2017) 7:6522. 10.1038/s41598-017-03262-2 28747771 PMC5529579

[38] Mukherjee R Kanti Barman P Kumar Thatoi P Tripathy R Kumar Das B Ravindran B. Non-Classical monocytes display inflammatory features: validation in sepsis and systemic lupus erythematous. Sci Rep. (2015) 5:13886. 10.1038/srep13886 26358827 PMC4566081

[39] Fraineau S Monvoisin A Clarhaut J Talbot J Simonneau C Kanthou C. The vitamin K-dependent anticoagulant factor, protein S, inhibits multiple VEGF-A-induced angiogenesis events in a Mer- and SHP2-dependent manner. Blood (2012) 120L5073–83. 10.1182/blood-2012-05-429183 23065156

[40] Diamond MS Springer TA. A subpopulation of Mac-1 (CD11b/CD18) molecules mediates neutrophil adhesion to ICAM-1 and fibrinogen. J Cell Biol. (1993) 120:545–56. 10.1083/jcb.120.2.545 7678422 PMC2119505

[41] Kowal J Arras G Colombo M Jouve M Morath JP Primdal-Bengtson B. Proteomic comparison defines novel markers to characterize heterogeneous populations of extracellular vesicle subtypes. Proc Natl Acad Sci USA. (2016) 113:E968–77. 10.1073/pnas.1521230113 26858453 PMC4776515

[42] Buzas EI Toth EA Sodar BW Szabo-Taylor KE. Molecular interactions at the surface of extracellular vesicles. Semin Immunopathol. (2018). 10.1007/s00281-018-0682-0 29663027 PMC6208672

[43] Raposo G Nijman HW Stoorvogel W Liejendekker R Harding CV Melief CJ. B lymphocytes secrete antigen-presenting vesicles. J Exp Med. (1996) 183:1161–72. 10.1084/jem.183.3.1161 8642258 PMC2192324

[44] Martinez-Lorenzo MJ Anel A Gamen S Monle NI Lasierra P Larrad L. Activated human T cells release bioactive Fas ligand and APO2 ligand in microvesicles. J Immunol. (1999) 163:1274–81. 10415024

[45] Monleon I Martinez-Lorenzo MJ Monteagudo L Lasierra P Taules M Iturralde M. Differential secretion of Fas ligand- or APO2 ligand/TNF-related apoptosis-inducing ligand-carrying microvesicles during activation-induced death of human T cells. J Immunol. (2001) 167:6736–44. 10.4049/jimmunol.167.12.6736 11739488

[46] Groot Kormelink T Mol S de Jong EC Wauben MHM. The role of extracellular vesicles when innate meets adaptive. Semin Immunopathol. (2018) 40:439–52. 10.1007/s00281-018-0681-1 29616308 PMC6208666

[47] van Niel G D'Angelo G Raposo G. Shedding light on the cell biology of extracellular vesicles. Nat Rev Mol Cell Biol. (2018) 19:213–28. 10.1038/nrm.2017.125 29339798

[48] Ravichandran KS. Find-me and eat-me signals in apoptotic cell clearance: progress and conundrums. J Exp Med. (2010) 207:1807–17. 10.1084/jem.20101157 20805564 PMC2931173

[49] Hochreiter-Hufford A Ravichandran KS. Clearing the dead: apoptotic cell sensing, recognition, engulfment, and digestion. Cold Spring Harb Perspect Biol. (2013) 5:a008748. 10.1101/cshperspect.a008748 23284042 PMC3579390

[50] Segawa K Nagata S. An Apoptotic 'Eat Me' Signal: Phosphatidylserine Exposure. Trends Cell Biol. (2015) 25:639–50. 10.1016/j.tcb.2015.08.003 26437594

[51] Ariel A Ravichandran KS. 'This way please': apoptotic cells regulate phagocyte migration before and after engulfment. Eur J Immunol. (2016) 46:1583–6. 10.1002/eji.201646505 27345468

[52] Patel AA Zhang Y Fullerton JN Boelen L Rongvaux A Maini AA. The fate and lifespan of human monocyte subsets in steady state and systemic inflammation. J Exp Med. (2017) 214:1913–23. 10.1084/jem.20170355 28606987 PMC5502436

[53] Ziegler-Heitbrock L Hofer TP. Toward a refined definition of monocyte subsets. Front Immunol. (2013) 4:23. 10.3389/fimmu.2013.00023 23382732 PMC3562996

[54] Zizzo G Hilliard BA Monestier M Cohen PL. Efficient clearance of early apoptotic cells by human macrophages requires M2c polarization and MerTK induction. J Immunol. (2012) 189:3508–20. 10.4049/jimmunol.1200662 22942426 PMC3465703

[55] Scott RS McMahon EJ Pop SM Reap EA Caricchio R Cohen PL. Phagocytosis and clearance of apoptotic cells is mediated by MER. Nature (2001) 411:207–11. 10.1038/35075603 11346799

[56] Lishko VK Yakubenko VP Ugarova TP Podolnikova NP. Leukocyte integrin Mac-1 (CD11b/CD18, alphaMbeta2, CR3) acts as a functional receptor for platelet factor 4. J Biol Chem. (2018) 293:6869–82. 10.1074/jbc.RA117.000515 29540475 PMC5936813

