# Supplementary material for: Differential Interaction of Platelet-Derived Extracellular Vesicles With Circulating Immune Cells: Roles of TAM Receptors, CD11b, and Phosphatidylserine
Source: Front Immunol. 2018 Dec 11;9:2797. doi: 10.3389/fimmu.2018.02797 (PMC6297748; doi:10.3389/fimmu.2018.02797)
Supplement: Supplementary file 1 [file Data_Sheet_1.pdf]

Supplementary Material

Differential Interaction of Platelet-Derived Extracellular Vesicles with Circulating Immune Cells: Roles of TAM Receptors, CD11b, and Phosphatidylserine

Birgit Fendl[1], Tanja Eichhorn[1], René Weiss[1], Carla Tripisciano[1], Andreas Spittler[2], Michael B. Fischer[1], Viktoria Weber[1*]

[1]Christian Doppler Laboratory for Innovative Therapy Approaches in Sepsis, Department for Biomedical Research, Danube University Krems, Krems, Austria

[2]Core Facility Flow Cytometry & Surgical Research Laboratories, Medical University of Vienna, Vienna, Austria

*** Correspondence:** Prof. Dr. Viktoria Weber: viktoria.weber@donau-uni.ac.at

Supplementary Table S1. Antibodies used for flow cytometry, confocal microscopy, blocking experiments, and Western Blotting. n.s., clone not specified.

Flow Cytometry

Antigen	Clone	Marker for	Fluorochrome	Abbreviation	Supplier
CD11b	CBRM1/5	activated CD11b	Fluorescein Isothiocyanate	FITC	BioLegend
CD14	RMO52	monocytes	Phycoerythrin	PE	Beckman Coulter
CD14	RMO52	monocytes	Allophycocyanin Alexa Fluor 750	APC AF750	Beckman Coulter
CD16	3G8	monocytes	Phycoerythrin Cyanin 5.1	PC5	Beckman Coulter
CD41	P2	platelets	Phycoerythrin Cyanin 7	PC7	Beckman Coulter
CD45	J33	leukocytes	Pacific Blue	PB	Beckman Coulter
CD66b	80H3	granulocytes	Allophycocyanin	APC	Beckman Coulter
CD235a	HIR2 (GA-R2)	erythrocytes	Allophycocyanin	APC	eBioscience
Axl	108724	Axl	Alexa Fluor 488	AF488	R&D Systems
lactadherin	-	phosphatidylserine	Fluorescein Isothiocyanate	FITC	Haematologic Technologies, Inc.
MERTK	590H11G1E3	Mer	Phycoerythrin Cyanin 7	PC7	BioLegend
TIM4	9F4	TIM4	Phycoerythrin	PE	BioLegend
Tyro3/Dtk	96201	Tyro3	Phycoerythrin	PE	R&D Systems

Confocal Microscopy

Antigen	Clone	Marker for	Fluorochrome	Abbreviation	Supplier
CD14	RMO52	monocytes	Phycoerythrin	PE	Beckman Coulter
CD41	MEM06	platelets	Alexa Fluor 488	AF488	Abcam
CD45	J33	leukocytes	Pacific Blue	PB	Beckman Coulter
CD66b	REA306	granulocytes	Phycoerythrin	PE	Miltenyi
lactadherin		phosphatidylserine	Alexa Fluor 647	AF647	Haematologic Technologies, Inc.

Blocking Experiments

Antigen	Clone	Supplier
CD11b	CBRM1/5	BioLegend
Axl	n.s.	Bio-Rad Antibodies
	polyclonal	R&D Systems
Mer	125508	R&D Systems
	Y323	Abcam
	125518	R&D Systems
Tyro3	96201	R&D Systems
	polyclonal	R&D Systems

Western Blot

Antigen	Clone	Supplier
Axl	polyclonal	R&D Systems
Mer	Y323	Abcam
Tyro3	polyclonal	R&D Systems
CD63	Ts63	Invitrogen
CD81	1.3.3.22	Invitrogen
Alix	OTI1A4	Bio-Rad Antibodies
αActinin-1	H-2	Santa Cruz

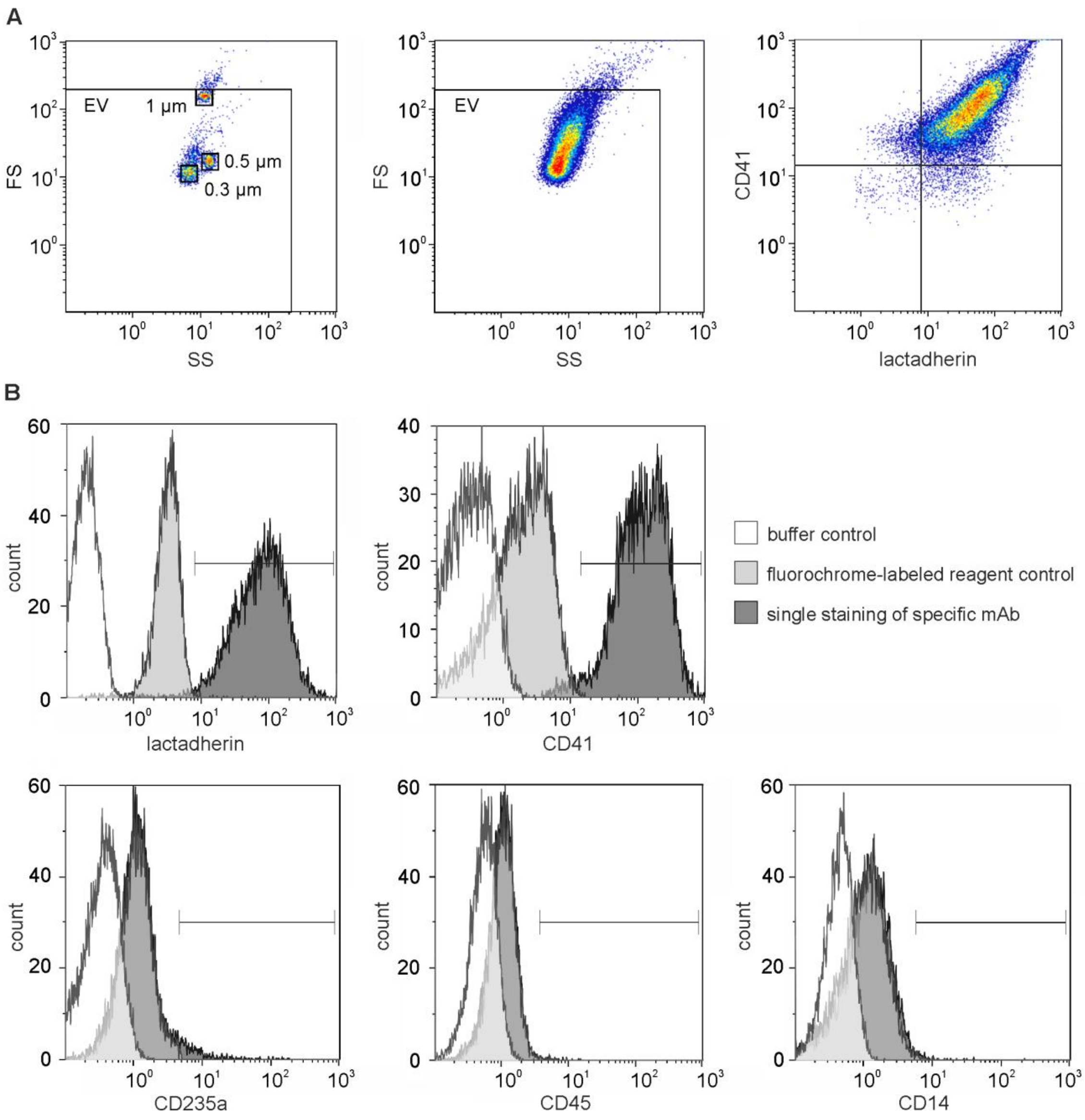

Supplementary Figure S1. Calibration and controls for the flow cytometric characterization of extracellular vesicles from platelet concentrate. (A) Flow cytometric characterization was performed on a Gallios Flow Cytometer (Beckman Coulter) after calibration with fluorescent-green silica particles (0.3, 0.5, and 1.0 µm), and the EV gate was set below the 1 µm bead cloud as described in the Methods section (left panel). Staining of platelet-derived EVs was performed with lactadherin as marker for phosphatidylserine in combination with an anti-CD41 monoclonal antibody (mAb) as platelet marker. A representative forward scatter *vs*. side scatter (FS *vs*. SS) dot plot (middle panel) as well as a CD41 *vs*. lactadherin dot plot (right panel) are shown. **(B)** The respective buffer controls, fluorochrome labeled reagent controls, and single stainings are shown (upper panel). To assess the presence of EVs derived from other blood cells, CD235a was used as a red blood cell marker, CD45 as leukocyte marker, and CD14 was used as monocyte marker (lower panel). Bars indicate positive expression.

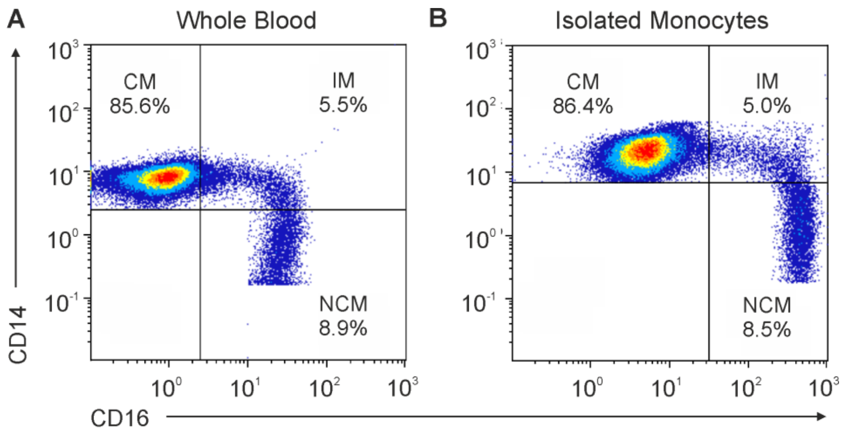

C

	Whole Blood	Isolated Monocytes
Classical Monocytes	85.6±2.2%	86.4±3.4%
Intermediate Monocytes	5.5±2.2%	5.0±1.5%
Non-Classical Monocytes	8.9±2.6%	8.5±2.1%

Supplementary Figure S2. Flow cytometric characterization of monocyte subsets in whole blood and after monocyte isolation. Monocyte subsets were identified based on their expression patterns of CD14 and CD16 either directly in whole blood or after isolation by density gradient centrifugation and negative depletion as described in the Methods section to discriminate classical (CM), intermediate (IM), and non-classical (NCM) monocytes. **(A)** Distribution of monocyte subsets in whole blood; **(B)** monocyte subset distribution in freshly isolated monocytes. **(C)** Summary of monocyte subset distribution (*n*=*7*). Data are indicated as mean ± standard deviation.

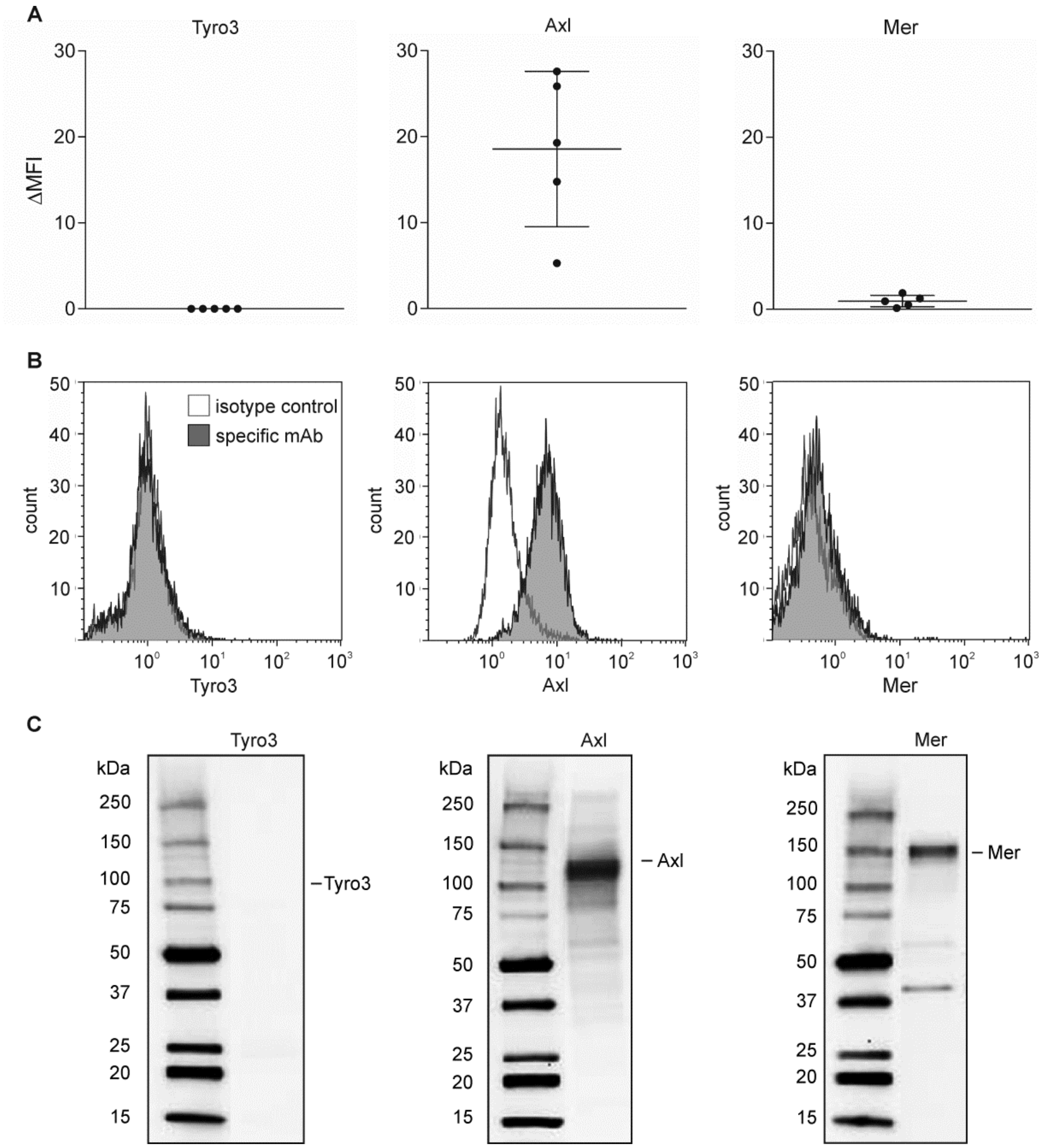

Supplementary Figure S3. Expression of TAM receptors on endothelial cells. (A) TAM receptor expression on human umbilical vein endothelial cells (HUVEC) was assessed by flow cytometry as described in the Methods section. Endothelial cells were labeled with Tyro3-PE, Axl-AF488, or Mer-PC7 monoclonal antibodies (mAb), specified in Table S1 (*n=5*). ΔMFI refers to the difference in mean fluorescence intensity between the fluorochrome-labeled specific antibodies and the corresponding isotype controls. **(B)** Representative histograms showing the specific receptor expression (grey) and the respective isotype controls (white). **(C)** Detection of Tyro3, Axl, and Mer on HUVEC using Western Blotting. Ten microgram of protein were loaded per lane.

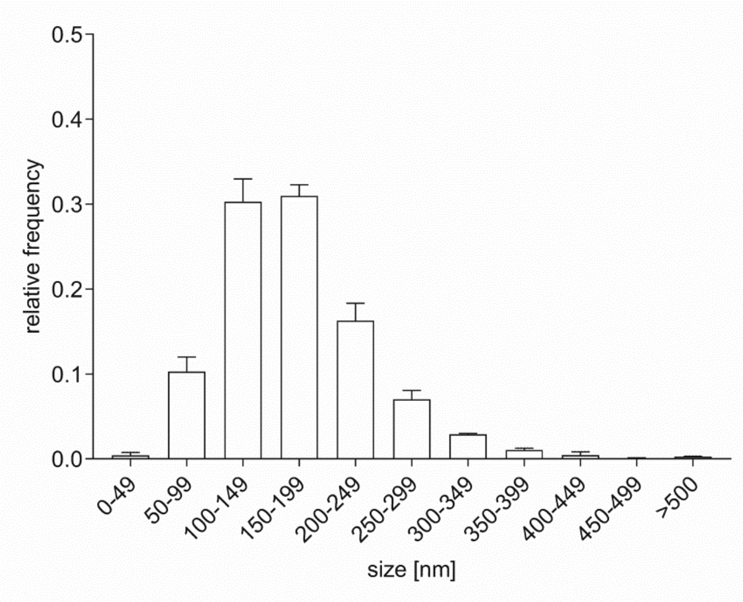

Supplementary Figure S4. Size distribution of platelet-derived extracellular vesicles. The size distribution of platelet-derived EVs (EV fraction I, obtained by centrifugation at 20,000 g as described in the Methods section) was analyzed using nanoparticle tracking analysis (Zeta View, Particle Metrix, Inning, Germany). Platelet-derived EVs were diluted to a final concentration of 0.6 µg/ml in $PBS^{-/-}$, and 1 ml of sample was injected into the flow cell. Measurement was performed at 22°C, using the following settings: 80 (sensitivity), 1000 (maximal area), 5 (minimal area), and 25 (brightness). Data were acquired in one cycle of measurement over 11 positions. The software ZetaView 8.04.02. was used for data analysis.

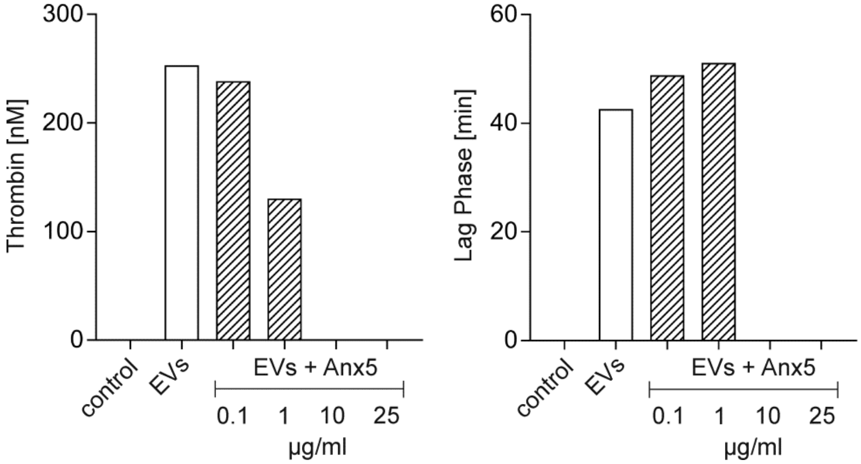

Supplementary Figure S5. Effect of phosphatidylserine blockade with Anx5 on thrombin generation induced by extracellular vesicles. The thrombogenicity of platelet-derived EVs was monitored using a thrombin generation assay as described in the Methods section. Pre-incubation of EVs with Anx5 decreased thrombin generation in a dose-dependent manner, indicating efficient masking of phosphatidylserine (*n=2*). Vesicle-free plasma served as control. Anx5, annexin V; EVs, extracellular vesicles.

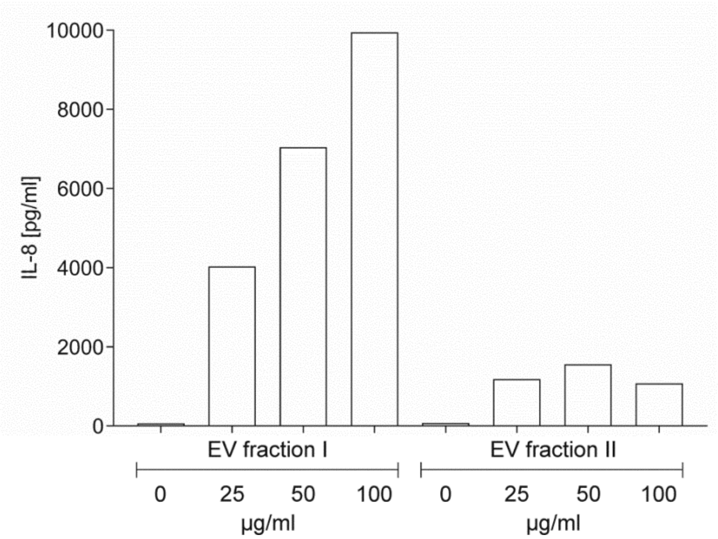

Supplementary Figure S6. Stimulation of monocytes with isolated extracellular vesicles. Monocytes were isolated from human whole blood by density gradient centrifugation and negative depletion as described in the main manuscript (section Monocyte and Granulocyte Isolation). Cells were seeded at a concentration of 5 x 10^4 per 200 µl in RPMI serum free medium in a 96-well flat bottom plate and were stimulated with increasing concentrations of EV fractions I and II (isolated from non-activated platelets as described in the main manuscript, section Isolation and Characterization of Extracellular Vesicles) for 8 h at 37°C in humidified atmosphere. Cell free supernatant was collected by centrifugation, and IL-8 was quantified by bead array assay (Bio-Plex Pro, Bio-Rad Laboratories). Monocytes stimulated with EV fraction I secreted IL-8 in a concentration-dependent manner.
